# C9ORF72 expression and cellular localization over mouse development

**DOI:** 10.1186/s40478-015-0238-7

**Published:** 2015-09-25

**Authors:** Rachel A K Atkinson, Carmen M. Fernandez-Martos, Julie D. Atkin, James C. Vickers, Anna E. King

**Affiliations:** Wicking Dementia Research and Education Centre, Faculty of Health, University of Tasmania, Hobart, Tasmania Australia; Australian School of Advanced Medicine, Macquarie University, North Ryde, New South Wales Australia

**Keywords:** C9ORF72, FTLD, ALS

## Abstract

**Introduction:**

A majority of familial frontotemporal lobar dementia and amyotrophic lateral sclerosis cases are associated with a large repeat expansion in a non-coding region of the *C9ORF72* gene. Currently, little is known about the normal function and the expression pattern of the C9ORF72 protein. The aims of this study were to characterize the expression pattern and cellular localization of the three reported mouse isoforms of *C9orf72*, over a developmental time-course in primary cultured cortical neurons and brain tissue from C57BL/6 mice.

**Results:**

We demonstrated that the different isoforms of C9ORF72 at the mRNA and protein level undergo alterations in expression during development and into adulthood. Cellular fractionation and immunofluorescence demonstrated that levels of nuclear and cytoplasmic expression of isoforms changed significantly over the time course. Additionally, immunofluorescence studies showed C9ORF72 labeling as puncta throughout neurons, extending beyond the microtubule cytoskeleton into actin-rich structures such as filopodia and growth cones. Finally, synaptosome preparations demonstrated the presence of C9ORF72 isoform 1 in synaptic-rich fractions from adult mouse brain.

**Conclusion:**

In summary, the presence of C9ORF72 as puncta and within synaptic-rich fractions may indicate involvement at the synapse and differential expression of isoforms in nuclei and cytoplasm may suggest distinct roles for the isoforms. Determining the physiological role of C9ORF72 protein may help to determine the role it plays in disease.

**Electronic supplementary material:**

The online version of this article (doi:10.1186/s40478-015-0238-7) contains supplementary material, which is available to authorized users.

## Introduction

Frontotemporal lobar dementia (FTLD) and amyotrophic lateral sclerosis (ALS) are progressive neurodegenerative disorders, which due to their overlapping features, are now thought to represent two ends of a disease spectrum [17]. In 2011, two independent groups identified the largest genetic cause of FTLD and ALS as a repeat expansion of the hexanucleotide sequence GGGGCC in the *C9ORF72* gene [4, 25]. This expansion occurs in a non-coding region of chromosome 9. It is currently unknown how the repeat expansion contributes to FTLD and ALS, although several mechanisms have been proposed, including potential unconventional translation of the repeated sequence (repeat-associated non-ATG initiated translation) leading to intracellular accumulations of dipeptide repeat proteins [1, 23], and the sequestration of RNA binding proteins into RNA foci, causing RNA dysfunction [4, 27]. Alternatively, the hexanucleotide expansion may result in haploinsufficiency due to reduced expression of C9ORF72 transcripts [2, 4, 5, 33, 34, 37].

While pathological features of *C9ORF72-*associated disease, such as TDP-43 aggregates, dipeptide repeat protein expression and RNA foci, are under intense investigation regarding their role in disease, to date, less attention has been paid to the normal expression and function of the encoded protein, C9ORF72. Elucidating the expression, localization and function of this protein in neural cells may contribute further to knowledge regarding how the repeat expansion is associated with neurodegenerative changes.

**Fig. 1:**
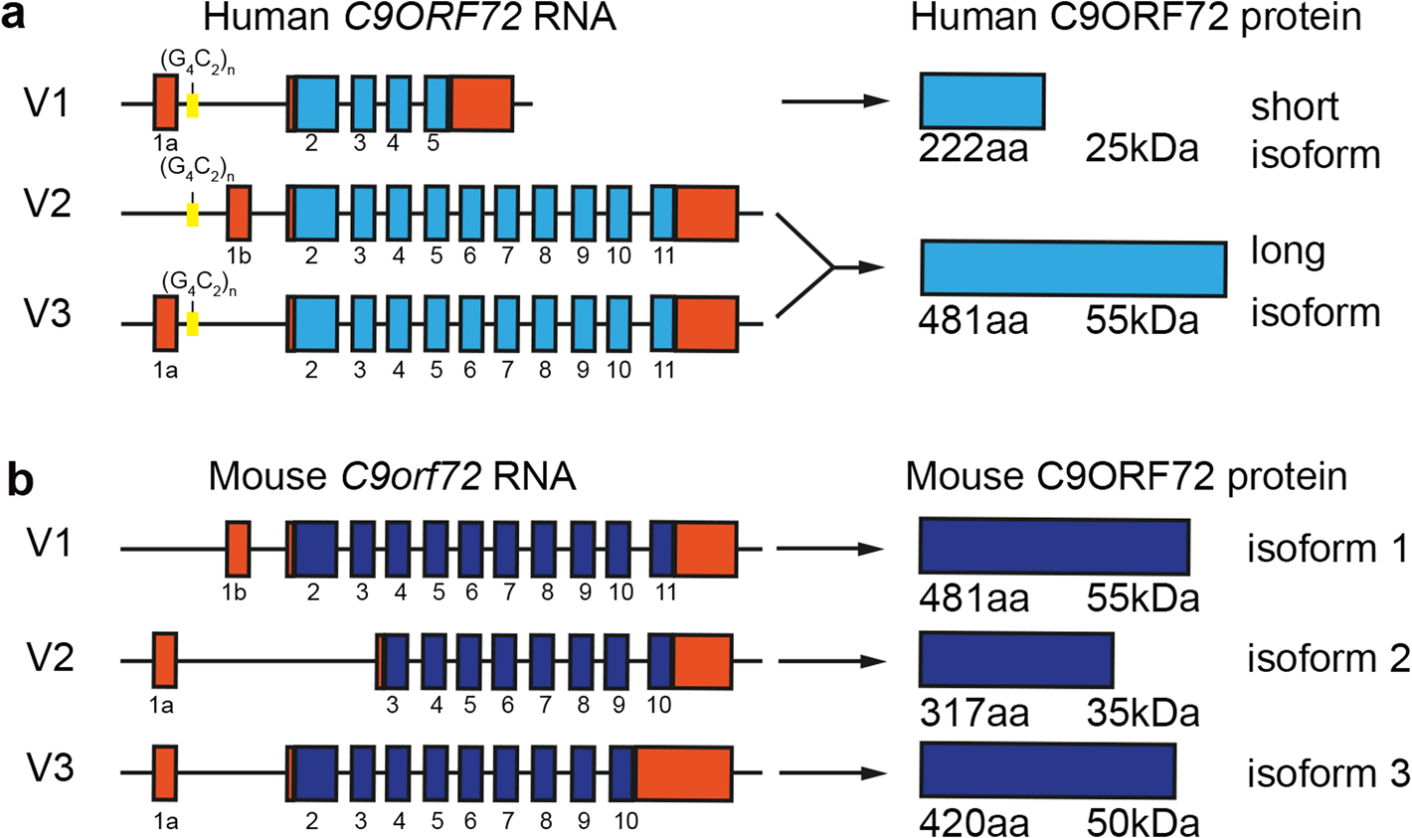
Schematic overview of human and mouse *C9ORF72* transcripts and encoded proteins. Protein coding regions for transcript variants (V1 to 3) are indicated in *light blue* for human (**a**) and *dark blue* for mouse (**b**) as well as size of encoded proteins. Non-coding regions are indicated in *red* and location of the G_4_C_2_ repeat expansion in *yellow*.

In humans, alternative splicing of three RNA transcript variants from the *C9ORF72* gene produces two different isoforms of the C9ORF72 protein (Fig. 1a) [25]. Transcript variants 1 and 3 encode a 481 amino acid protein and variant 2 encodes a 222 amino acid protein [4]. In mice, there are 3 protein-coding regions reported of 481 (isoform 1), 420 (isoform 2) and 317 (isoform 3) amino acids, likely encoding at least 3 different protein isoforms (Fig. 1b). However, the roles of the encoded proteins have not been well characterized. We have previously demonstrated a role for C9ORF72 in trafficking [7] which was in line with previous studies [16]. C9ORF72 is involved in endosomal trafficking via Rab-dependent pathways. Rab proteins are part of the Rab-GDP/GTP exchange factor family (Rab-GEF) (as reviewed in [29]) that mediate all membrane trafficking events between organelles. We provided the first experimental evidence for this, when we established that C9ORF72 regulates endocytosis and autophagy [7].

Other studies have examined the expression of the *C9orf72* gene using a transgenic mouse model harboring a targeted LacZ insertion [32]. This study observed *C9orf72* in neuronal and non-neuronal cells within the central nervous system (CNS). Recently, the effect of ablating the 3 isoforms of C9ORF72 protein from neurons and glia has been examined, demonstrating a reduction in body weight but no motor neuron degeneration or motor deficits [15]. This suggests that complete lack of C9ORF72 throughout development and adulthood is not sufficient to cause a motor neuron disease phenotype in mice.

Several studies have examined the expression of C9ORF72 in human tissue [3, 4, 11, 13, 28, 30] and cell lines [11, 25] using a variety of commercial antibodies. However, there has been a lack of consensus about the localization of C9ORF72 across these studies. Some investigations have described coarse punctate expression within the hippocampus, suggestive of synaptic terminals [3, 13, 26, 28]. Recently, Xiao and colleagues [37] generated antibodies specific to the two human C9ORF72 isoforms. They demonstrated diffuse cytoplasmic and ‘speckled’ localization of the long isoform, as well as localization of the short isoform to the nuclear membrane. This is in line with our previous investigation which demonstrated a nuclear and punctate pattern of expression (typical of vesicles) of C9ORF72 *in vitro* in both SH-SY5Y cells and in primary cultured cortical neurons [7].

The current study examined the expression of C9ORF72 in the mouse CNS over development *in vivo* and *in vitro* in order to provide information about its expression and cellular localization during neurite outgrowth, neuron maturation and synapse formation. This investigation demonstrated that expression of C9ORF72 mRNA and protein differ over a developmental time course, are expressed in both nuclear and cytoplasmic fractions in an isoform specific manner, and that the large isoform may be present in synaptic fractions.

## Materials and methods

### Animals

C57BL/6 mice were utilized in this study. All experiments involving animals were approved by the University of Tasmania Animal Ethics Committee (A12780) and were in accordance with the Australian Guidelines for the Care and Use of Animals for Scientific Purposes.

### Tissue preparation

For molecular biology analysis, combined neocortical and hippocampal tissue was harvested from mice at embryonic day (E) 18, postnatal day (P) 1, P7, P14, P28 and P56 (for western blot) (*n* = 4 mice per time-point) and P1, P7 and P56 (for real time qPCR) (*n* = 4 mice per time-point). Tissue was processed as previously described [18].

For immunohistochemical analysis, animals were terminally anaesthetized with sodium pentobarbitone (140 mg/kg) and transcardially perfused with 4 % paraformaldehyde. Brains were immediately dissected, post-fixed overnight in paraformaldehyde and then cryoprotected as previously described [19]. Serial 40 μm coronal sections were cut on a cryostat (Leica CM 1850). For each mouse at each time point, four regularly spaced sections were examined from the rostral to caudal cortex corresponding to bregma 0.98 mm to −1.82 mm (in adult tissue) according to the stereotaxic mice atlas [10]. Antigen retrieval was carried out prior to immunohistochemistry using citric acid, pH 6.0, in a pressure cooker for 14 min.

### Protein extraction and western blot analysis

Protein from combined cerebral cortex and hippocampus was extracted with RIPA Buffer (Sigma Aldrich) containing a cocktail of protease inhibitors (Roche). Protein extract was then placed at 4 °C for 30 min, centrifuged at 13,000 rpm for 20 min and supernatant stored at −80 °C.

Denatured proteins samples (15 μg) from each time-point were electrophoresed into 10 % SDS-PAGE gels (BioRad), transferred to PVDF membranes (BioRad) and incubated in primary antibodies overnight (Table 1). A corresponding anti-rabbit or anti-mouse horseradish peroxidase (HRP)-conjugated secondary antibody (1:7000; Amersham) was used, as described previously [18]. GAPDH (1:7000, Millipore) was used as a loading control and band intensity was measured as the integrated intensity using ImageJ software (v1.4; NIH), and normalized with respect to the loading controls. Three experimental repeats were carried out.

**Table 1:** List of qPCR primers

Gene name	Forward primer	Reverse primer	Accession number
*C9orf72* isoform 1	5’- CCCACCATCTCCTGCTGTTG-3’	5’-GTAAGCAAAGGTAGCCGCCA-3’	NM_001081343.1
*C9orf72* isoform 2	5’- TGGAAGATCAGGGTCAGAGT-3’	5’- GCAAGCAGCTCCATTACAGG-3’	XM_006538294.1
*C9orf72* isoform 3	5’-CTTTCCTTGCACAGTTCCTCC-3’	5’- TCATCCTCGATGTACTTGATTAGTG-3’	XM_006538292.2

Primers used for qPCR analysis of the 3 *C9orf72* isoforms including primer sequence (forward and reverse sequence respectively) and GenBank accession number

### Nuclear and cytoplasmic fractionation

Nuclear and cytoplasmic protein extractions were prepared from right hemispheres (excluding olfactory bulbs and cerebellum) of fresh P1, P7, P56 brains using the NE-PER kit (Thermo Fisher Scientific) according to manufacturer instructions. Denatured protein samples were electrophoresed as described above. Fraction purity was confirmed by labeling with HDAC2 (1:700; Abcam) for nuclear fractions and GAPDH (as above) for cytoplasmic fractions. Membranes were also incubated with C9ORF72 antibody (as above). Densitometry analysis of bands was carried out using ImageJ. Results were normalized to total protein. Three experimental repeats were carried out.

### Synaptosome preparation

Synaptosomes were prepared as described previously [6, 22] with some modifications. Briefly, P56 mice (*n* = 4) were anaesthetized and perfused with sucrose buffer (0.32 M sucrose, 1 mM ethylenediaminetetraacetic acid, 5 mM dithiothreitol, pH 7.4). Whole brains were harvested and homogenized at 4 °C with a teflon-glass homogenizer using 12 strokes with 9:1 ratio of sucrose buffer supplemented with a protease cocktail inhibitor (Roche) to 1 g of tissue. Homogenate was centrifuged at 1000xg for 10 min at 4 °C. The resulting pellet containing mostly nuclei was removed and the supernatant was layered onto a discontinuous gradient consisting of 3, 10, 15 and 23 % (vol/vol) Percoll (GE Healthcare). Tubes were then centrifuged at 31,000xg for 8 min at 4 °C in a Sorvall WX Ultra90 (70.I TI rotor).

The contents of the resulting fractions have been characterized previously [6, 31]. The resulting purified fractions were collected and protein was extracted in RIPA buffer for western blotting. Denatured protein samples (15 μg) were electrophoresed as described above. Membranes were probed with C9ORF72 antibody, along with synaptic markers: synaptophysin (1:5000, Millipore), PSD-95 (1:1000, Abcam), GAD67 (1:2500, Millipore); and GFAP (1:1000, NeuroMAB) as a marker of glia.

### RNA isolation and RT-PCR analysis

Total RNA from combined cerebral cortex and hippocampus tissue at the time-points P1, P7 and P56 (*n* = 4 mice per time point) was isolated using the RNeasy Mini Kit (Qiagen), according to the manufacturer’s instructions and complementary DNA (cDNA) was synthesized from DNase-treated RNA (1 μg) as described previously [8].

To semi-quantitatively analyse *C9orf72* gene expression, quantitative PCR (qPCR) analysis was conducted as previously described [9]. Before relative quantification, *C9orf72* gene was subjected to a serial dilution assay to determine the optimum detection range of Ct values, with a Ct threshold of 35 for undetectable mRNA levels of expression. Relative quantitation of *C9orf72* mRNA isoforms per time point was performed using 25 ng of reverse-transcribed total RNA, 20 pmol/ml of both sense and antisense primers and the SYBR Green PCR master mix (Applied Biosystems) in a final reaction volume of 10 μl. The reactions were run on an LightCycler® 480 System (Roche) according to the manufacturer’s instructions. To standardize the amount of sample cDNA added to the reaction, amplification of endogenous control β-Actin (primer sequence obtained from Gonzalez-Fernandez and colleagues [12]) were included in a separate well as a real-time reporter. Primer efficiency was calculated (Additional file 1: Figure S1a), and at the end of each run, melting curve profiles were performed to confirm amplification of specific transcripts (Additional file 1: Figure S1b). Relative quantification for each gene was performed by the ∆∆Ct method [20].

All primers were designed using NCBI**/**Primer**-**BLAST software (Table 1). Primers were designed to amplify the different isoforms of the *C9orf72* mouse ortholog (*3111004O21Rik*). As *C9orf72* isoforms 2 and 3 are contained within isoform 1, fold change in the mRNA expression of isoform 2 and 3 were calculated as the increment with respect to the expression levels of isoform 1.

### Cell culture

Primary dissociated cortical cultures were prepared as previously described [14] using standard culture techniques with slight modifications. Briefly, neocortical tissue was harvested from E15.5 C57BL/6 mice and enzymatically dissociated in 0.0125 % trypsin for 4 min, prior to plating. Cells were plated onto poly L-lysine (Sigma Aldrich) coated 12 mm coverslips in 24 well plates at a density of 30,000 viable cells per coverslip. Cells were grown in an initial plating media consisting of Neurobasal^™^ medium (Gibco), 2 % B27 supplement, 10 % fetal calf serum (Gibco), 0.5 mM glutamine, 25 mM glutamate and 1 % antibiotic/antimycotic (Gibco). Medium was replaced on the following day with subsequent growth media consisting of initial media without the fetal calf serum and glutamate, and half the media was replenished weekly with fresh subsequent growth medium. Cultures were grown at 37 °C and 5 % CO_2_. Neurons were fixed with 4 % paraformaldehyde (Sigma Aldrich) at 1, 3, 7, 14 and 21 days *in vitro* (DIV) (*n* > 5 cultures per time-point).

### Immunofluorescence

Cultured cells and brain sections were washed 3x10 minutes in 0.01 M PBS followed by serum-free protein block (Dako) for 15 min at room temperature (RT). Immunofluorescence labeling was carried out for both cultured cells and brain tissue following standard procedures using antibodies against C9ORF72 (as above), β-III Tubulin (1:5000, Promega) and MAP2 (1:1000, Millipore) diluted in PBS with 0.6 % Triton-X-100 and incubated at RT overnight. Samples were incubated in secondary antibodies (AlexaFluor, Invitrogen Probes) for 2 h at RT, followed by incubation with the nuclear stain DAPI (5 μg/ml; Molecular Probes®, Life Technologies), for 5 min at RT. Immunoreactivity was visualized and captured using a Leica (Germany) DM BL2 upright fluorescence microscope. For the purpose of illustration, images were then adjusted for brightness and contrast using Adobe Photoshop CS6 (v 13).

Specificity of immunoreactivity was confirmed by two methods. Both brain sections and cultured cells were examined for non-specific labeling after processing without primary antibody. Additionally, tissue from P56 brain and seven DIV cultures were incubated with C9ORF72 antibody combined with seven times excess of C9ORF72 peptide (sc-138763 P; Santa Cruz).

### Statistical analyses

All statistical analysis was performed using GraphPad Prism software (version 6.0) and p-values with *p* < 0.05 (CI 95 %) considered significant. Values were reported as the mean ± standard error (SEM). Data from real time PCR studies were compared using a one-way ANOVA followed by a Tukey post-hoc and t-tests for a point-to-point comparison. Data from western blots were compared using a two-way ANOVA followed by Tukey or Sidak post-hoc comparisons.

## Results

### Cellular pattern of C9ORF72 protein changes over development of mouse cortex

This study utilized the commercially available anti-human C9ORF72 antibody raised against amino acid residues 165 to 215 of C9ORF72 protein, which is contained within the sequence of all three mouse C9ORF72 isoforms. We have previously shown a decrease in labeling in a western blot with the antibody following treatment with C9ORF72 short interfering RNA (siRNA) [7]. To further characterize the specificity of immunolabeling, the C9ORF72 antibody was preadsorbed with recombinant peptide. Immunolabeling of tissue sections from P56 mice (Additional file 2: Figure S2a) and cultured cortical neurons at 7 DIV (Additional file 2: Figure S2b) with preadsorbed C9ORF72 demonstrated that, relative to non-adsorbed antibody, there was a large reduction in immunolabeling (Additional file 2: Figure S2).

**Fig. 2:**
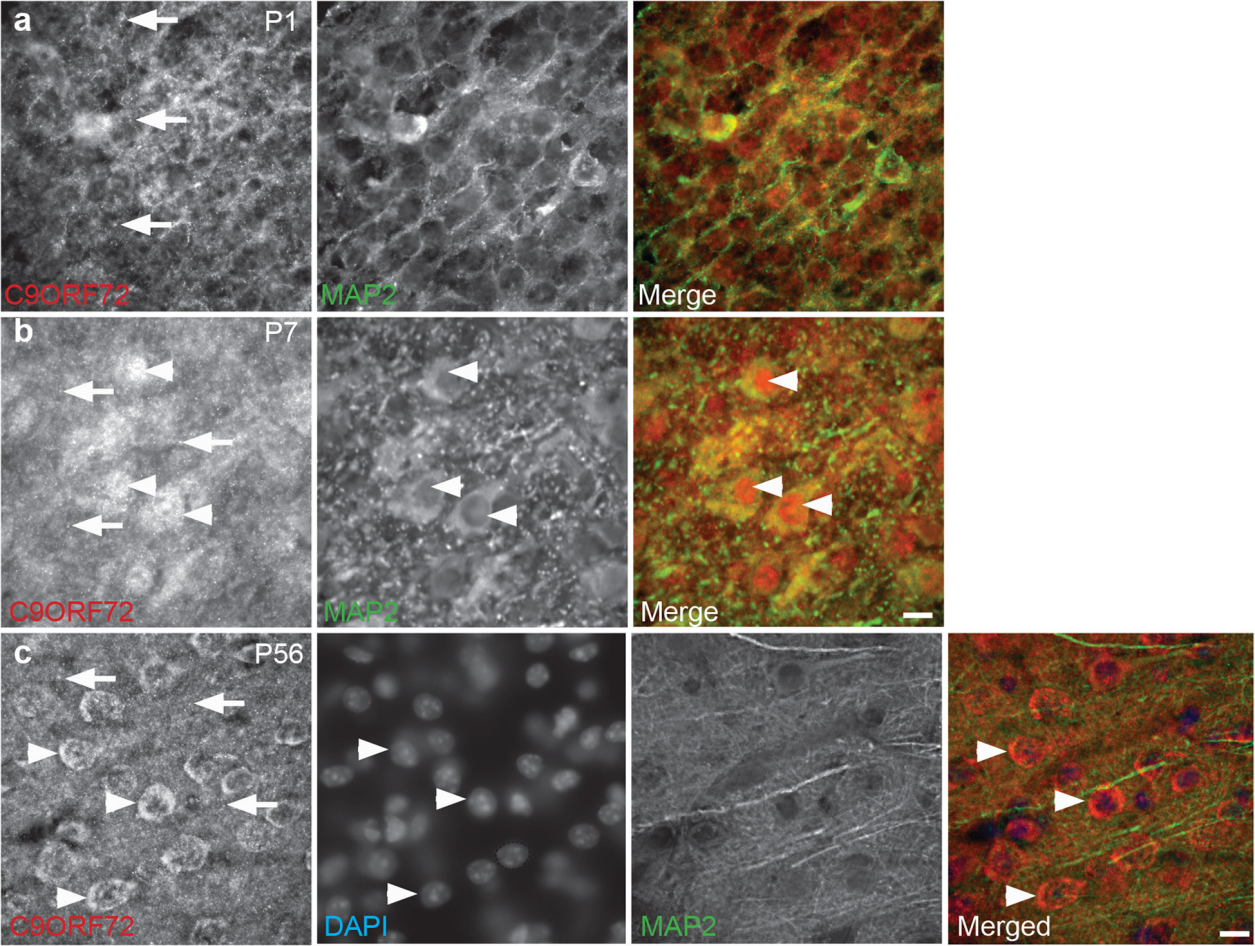
Localization of C9ORF72 during development *in vivo.* **a** At P1, C9ORF72 (red) had punctate localization throughout the neuropil (*arrows*). **b** At P7, C9ORF72 labeling was present within nuclei (*arrowheads)* of neuronal cells (MAP2, green) and as strong puncta within cytoplasm and neuropil (*arrows*). **c** At P56, C9ORF72 labeling was present in the neuropil as puncta (*arrows*) and was localized to the cytoplasm surrounding nuclei (DAPI, arrowheads) Scale bar: 10 μm

We next determined the expression or localization of C9ORF72 over a developmental time course from E18 to P56 in mice, which covers periods of neurite outgrowth and synapse development [24, 36]. To determine how C9ORF72 localization changes over development, 40 μm coronal tissue sections from mice at ages E18, P1, P14, P28 and P56 were immunolabeled with C9ORF72 antibody along with the neuronal somatodendritic marker, MAP2. At both E18 (data not shown) and P1, there was strong labeling for C9ORF72 in discrete puncta throughout the neuropil (Fig. 2a) but little somal immunoreactivity was present. At P7, there was distinct somal and nuclear expression, which was both diffuse and punctate in many MAP2-immunoreactive cells throughout the cortex and hippocampus (Fig. 2b), confirming the presence of C9ORF72 in neurons. At P14, P28 and P56, cytoplasmic labeling continued with apparent lower expression in nuclei (Fig. 2c). Punctate labeling was less distinct than at P1 and P7 (Fig. 2a and b).

### Temporal expression of C9ORF72 isoforms over development

**Fig. 3:**
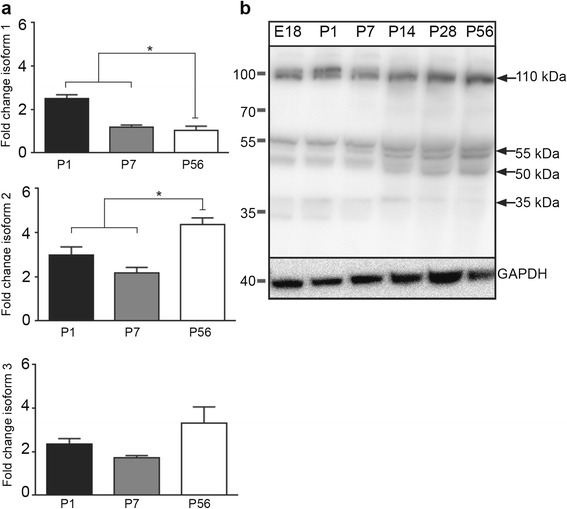
Expression of C9ORF72 isoforms over development. **a** Relative expression of *C9orf72* isoforms 1, 2 and 3 mRNA in combined cerebral cortex and hippocampus of C57Bl/6 mice. Isoform 1 was significantly (*p* < 0.05) higher at P1 compared to P7 and P56 and isoform 2 was significantly (*p* < 0.05) higher at P56 compared to P1 and P7. **b** Western blot of C9ORF72 expression in mouse tissue over development. Bands corresponding to reported isoforms of C9ORF72 were present at 55, 50 and 35 kDa (b). Additional bands at 110 and 50 kDa were also present. GAPDH was used as a loading control. Values represent mean ± standard error. *p < 0.05 P1 and P7 vs. P56

We evaluated the temporal mRNA expression pattern of *C9orf72* isoforms (*C9orf72- 1, 2* and *3*; Fig. 1). As shown in Fig. 3a, the mRNA encoding for all *C9orf72* isoforms were detected in combined cerebral cortex and hippocampus tissue at all time-points P1, P7 and P56. Isoform 1 was significantly (*p* < 0.05) higher at P1 compared to P7 and P56 (Fig. 3a) and, similarly, the expression of isoform 2 was significantly (*p* < 0.05) higher at P56 relative to the other time points tested (Fig. 3a). There were no changes in the mRNA expression levels of isoform 3 over development (Fig. 3a). Next, by western blot analysis, we evaluated the temporal protein expression of C9ORF72 protein-coding regions (481, 420 and 317 amino acids), which correspond to predicted protein size isoforms of approximately 55, 50 and 35 kDa. Western blots of combined cerebral cortex and hippocampus tissue demonstrated that the Santa Cruz C9ORF72 antibody labeled the three predicted protein isoforms at 55, 50 and 35 kDa at all the time points (Fig. 3b). Moreover, we also detected additional bands at 110 kDa. The identity of this band remains unknown.

### Subcellular localization of C9ORF72 over development

**Fig. 4:**
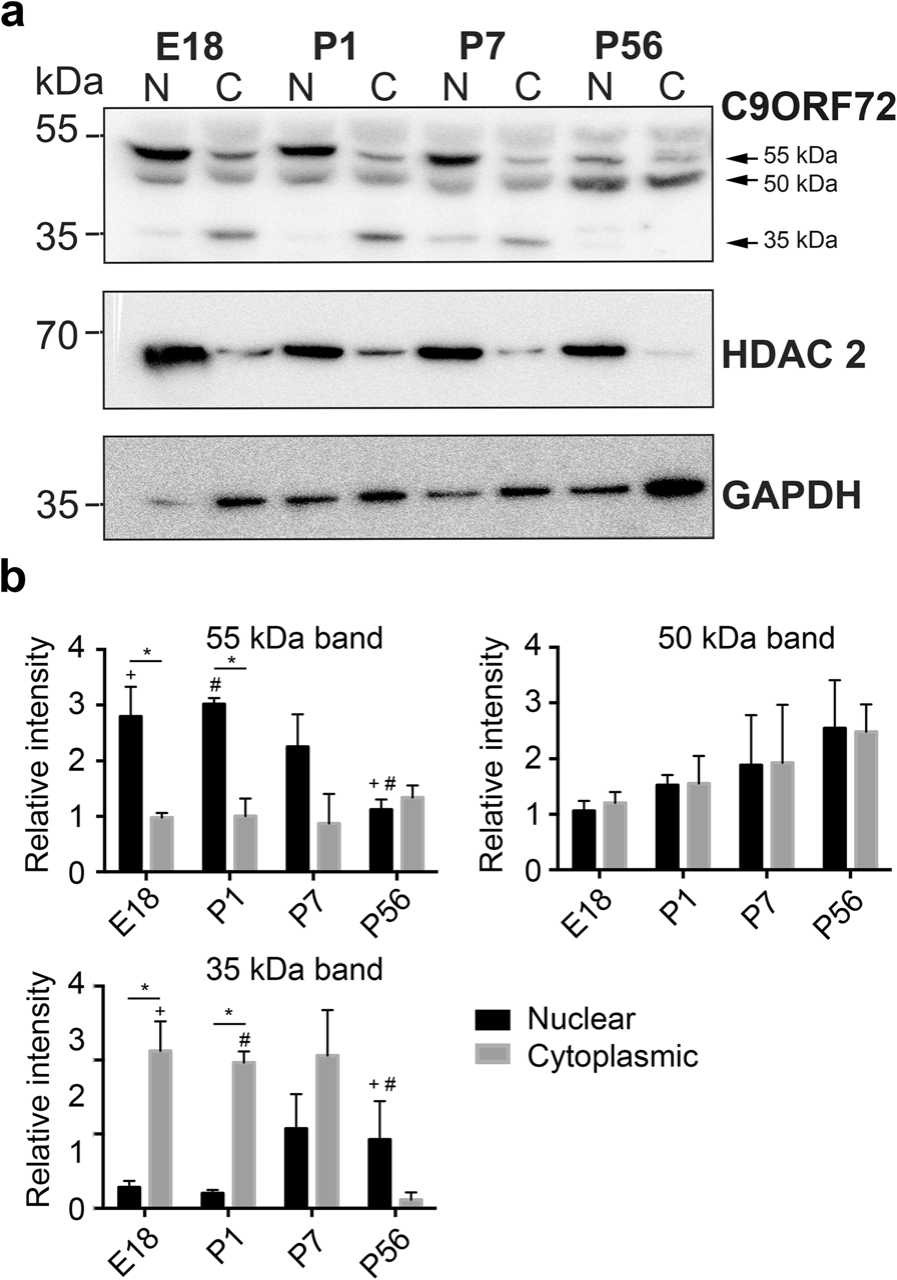
Expression of C9ORF72 in nuclear and cytoplasmic protein fractions over development. **a** Representative western blot of C9ORF72 in nuclear and cytoplasmic fractions from E18, P1, P7, and P56 mouse brain. HDAC2 and GAPDH were used to demonstrate nuclear and cytoplasmic fractions respectively. C9ORF72 isoforms were present at differing levels and in specific fractions throughout the time course. **b** Relative quantitation of C9ORF72 isoform expression in nuclear and cytoplasmic fractions over the time course. Overall, the 55 kDa protein was significantly (*p* < 0.05) increased within nuclear fractions compared to cytoplasmic fractions and specifically at E18 and P1 compared to P56 (*p* < 0.05). Within nuclear fractions, the 55 kDa protein was significantly (*p* < 0.05) increased at P1 compared to P56. There were no significant differences in nuclear and cytoplasmic expression for the 50 kDa band. Overall, the 35 kDa band was significantly (*p* < 0.05) increased within cytoplasmic fractions compared to nuclear fractions and specifically at E18 compared to P56 (*p*< 0.05). Within cytoplasmic fractions, the 35 kDa protein was significantly increased (*p* < 0.05) at E18, P1 and P7 compared to P56. Values represent mean ± standard error. *p < 0.05 nuclear vs. cytoplasmic at E18 and P1. + *p* < 0.05 E18 vs. P56 (nuclear for 55 kDa bad, cytoplasmic for 35 kDa band). # *p* < 0.05 P1 vs. P56 (nuclear for 55 kDa band, cytoplasmic for 35 kDa band)

To further investigate the differential nuclear and cytoplasmic localization of C9ORF72 over development *in vivo*, nuclear and cytoplasmic protein extractions were performed at E18, P1, P7 and P56. Purity of the nuclear and cytoplasmic extractions was confirmed with HDAC 2 and GAPDH antibodies with HDAC2 being higher in the nuclear samples and GAPDH being higher in the cytoplasmic samples (Fig. 4a). As in non-fractionated samples, western blot analysis of C9ORF72 showed protein bands at approximately 55, 50 and 35 kDa. The 55 kDa protein was significantly (*p* < 0.05) higher in the nuclear fractions compared to cytoplasmic fractions (Fig. 4b). Post hoc tests showed that at E18 and P1 there was significantly (*p* < 0.05) more of the 55 kDa protein in nuclear fractions compared to cytoplasmic fractions. Post hoc tests also showed that within nuclear fractions, there was significantly (*p*< 0.05) more 5 kDa protein at E18 compared to P56. There were no significant differences in cytoplasmic expression of the 55 kDa protein over the time course. There were also no differences in localization of the 50 kDa protein over the time course (Fig. 4b). The 35 kDa protein was significantly higher in the cytoplasmic fractions compared to nuclear fractions (*p* < 0.05) (Fig. 4b). Post hoc tests showed that, at E18 and P1, there was significantly (*p* < 0.05) more of the 55 kDa protein in cytoplasmic fractions compared to nuclear. Post hoc tests also showed that within cytoplasmic fractions there was significantly (*p* < 0.05) more 35 kDa protein at E18, P1 and P7 compared to P56. There were no significant differences in nuclear expression of the 35 kDa protein over the time course. These results suggest that C9ORF72 protein isoforms were differentially expressed in cellular compartments over development.

### C9ORF72 is present in synaptosome preparations

**Fig. 5:**
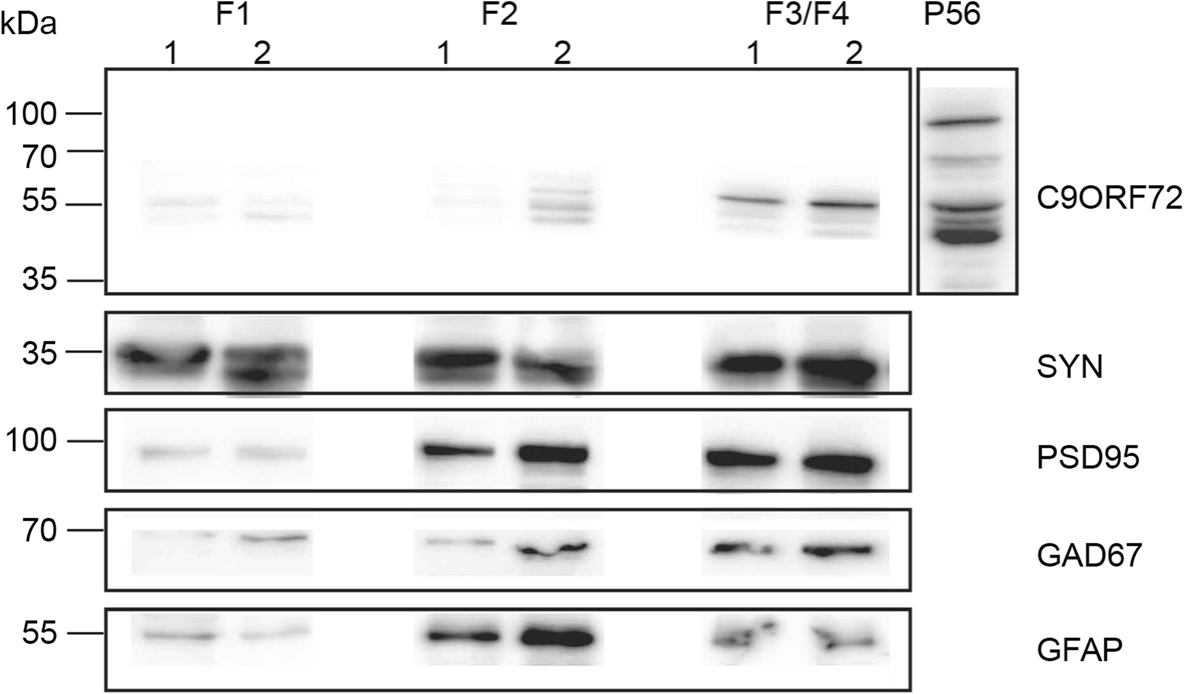
Expression of C9ORF72 in synaptosome preparations from mouse brains. Figure shows representative western blots with results from 2 animals for each marker (indicated by 1 and 2 on figure). The 55 kDa isoform of C9ORF72 was present in the combined F3/F4 fractions which contain synaptosomes. C9ORF72 expression was low in fraction F1, containing membranes, and F2 containing myelin, membranes and glia. Unfractionated brain at P56 was also included. Purity of fractions was determined by labeling with GFAP (F2) and synaptic markers, synaptophysin, PSD-95 and GAD67 (F3/F4)

Our results showed that C9ORF72 has punctate localization in the neuropil, however, it is unclear if it is present in synapses. To address this, we performed subcellular fractionation on P56 mouse brain tissue to isolate synaptosome fractions according to the methods of Dunkley, Jarvie and Robinson [6]. The content of each resulting fraction have been characterized previously [6, 31]. F1 contains unidentified membranous material [6], F2 contains predominantly re-sealed plasma membranes from glial cells [31]. F3 and F4 contain purified synaptosomes and these fractions were combined [6]. To confirm the purity of the fractions, western blotting was carried out with a range of antibodies. GFAP was used as a glia marker and was most abundant in F2 (Fig. 5). As expected, synaptophysin, PSD95 and GAD67 were most abundant within the F3/F4 fraction (Fig. 5). Only the 55 kDa protein band of C9ORF72 was observed within C9ORF72-positive fractions, and was most abundant within F3/F4 fractions where other synaptic proteins were found. It was also present at low levels in F2.

### C9ORF72 is expressed in nuclei and neurites of cultured cortical neurons

**Fig. 6:**
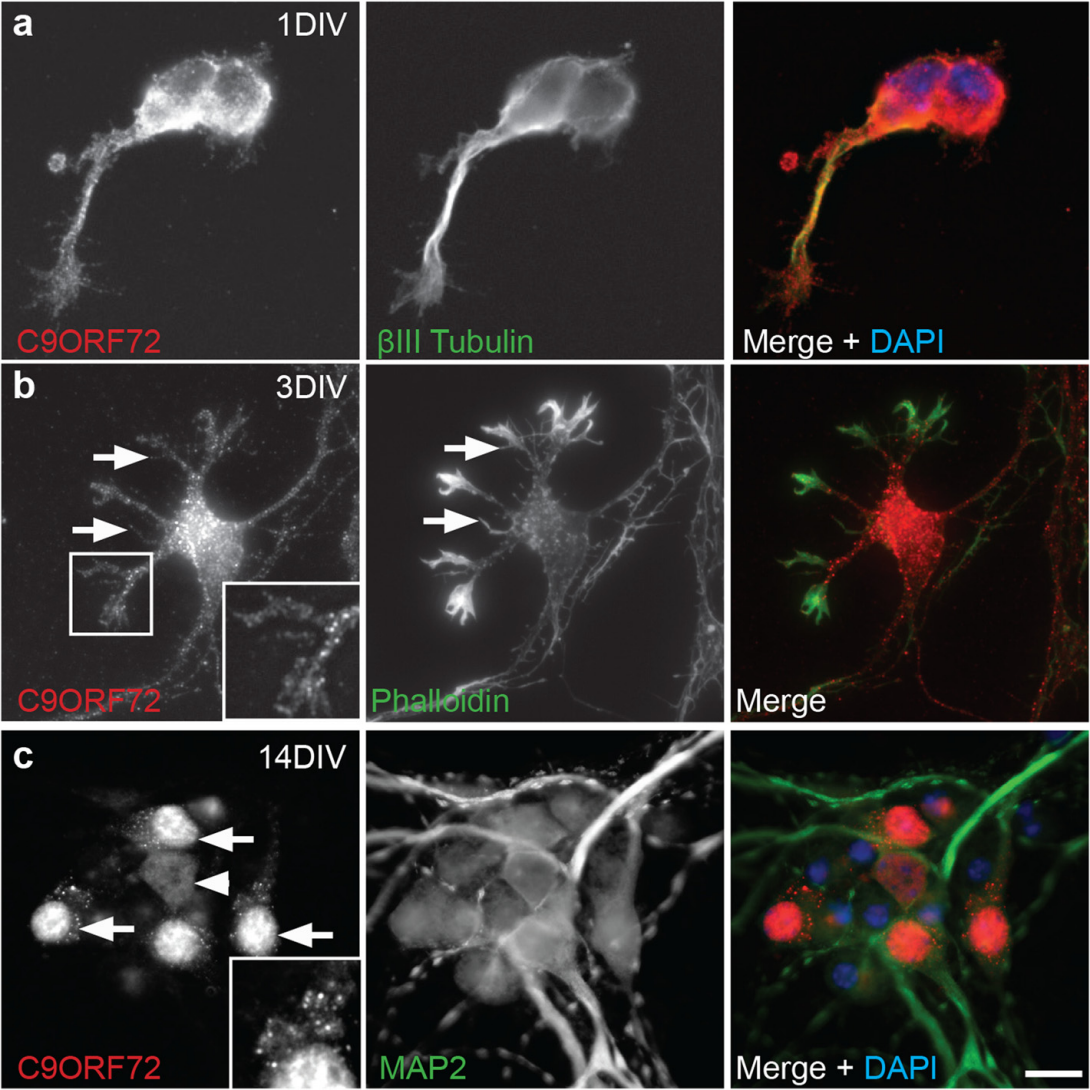
Localization of C9ORF72 over development *in vitro*. Immunofluorescence was carried out on primary cultured cortical neurons. **a** At 1 DIV, C9ORF72 (*red*) labeling was present within cell bodies, excluding nuclei (DAPI, *blue*) and punctate localization was present in neurites and growth cones (β-III tubulin, *green*). **b** Co-staining with phalloidin (*green*) at 3 DIV confirmed localization of C9ORF72 (*red*) labeling to growth cones and to filopodia (*arrows*). **c** At 14 DIV, C9ORF72 (*red*) was localized to nuclei of a population of neurons (*arrows*) but was less intensely expressed in nuclei of other neurons (*arrowhead*). Neurons indicated by MAP2 (*green*). Neurons with nuclear immunolabeling for C9ORF72 frequently had punctate somal localization of this protein. Inset (**c**) shows C9ORF72 labeling in nuclei and in puncta in surrounding cytoplasm. Scale bar: A, 2.5 μm; B, C, 10 μm

For a more detailed examination of the localization of C9ORF72 in neuronal soma and neurites, immunocytochemistry was performed in cultured cortical neurons fixed at 1, 3 and 7 DIV (during neurite outgrowth) and 14 and 21 DIV (during synaptogenesis and maturity). Neurons were labeled with C9ORF72 along with neuronal cytoskeletal markers βIII-tubulin and MAP2 and the F-actin stain, phalloidin. At 1 and 3 DIV, C9ORF72 labeling was present in the cell soma, excluding the nucleus, and throughout the neurites as demonstrated by co-labeling with βIII-tubulin (Fig. 6a). C9ORF72 also extended into the actin cytoskeleton, including within growth cones and filopodia extending from the soma and down the length of neurites, as demonstrated by co-labeling with phalloidin (Fig. 6b).

From 7 DIV, immunolabeling for C9ORF72 increased in the soma and a large proportion of cells had high nuclear expression of the protein, accompanied by bright puncta in the soma (data not shown). Similar cellular localization was observed at 14 DIV with bright vesicular labeled puncta more prominent in, but not restricted to, neurons with nuclear expression of C9ORF72 (Fig. 6c). A smaller proportion of neurons had more diffuse immunolabeling which was present in less intensely stained puncta in the cytoplasm and neurites (axons and dendrites, demonstrated by MAP2 co-labeling) in cells with nuclear and non-nuclear labeling. Immunolabeling of C9ORF72 was similar at 21 DIV. These results show that C9ORF72 was present throughout the microtubule cytoskeleton including throughout the axon, soma and dendritic arbor as well as within actin-rich structures such as growth cones and filopodia.

## Discussion and conclusions

In this study, we have examined the expression of C9ORF72 by multiple biochemical and molecular biological analyses conducted both *in vivo* and *in vitro*. Results from these investigations demonstrated that C9ORF72 undergoes alterations in cellular expression and localization throughout the time course analyzed, which may reflect differential expression of isoforms that are present in specific locations. Furthermore C9ORF72 is found in synaptic-rich cellular fractions.

In order to gain some insight into the function of C9ORF72 protein, we examined whether the expression level was altered throughout development. Neuronal development involves a number of different processes and therefore alterations in the expression or localization of proteins during development may indicate a role in these processes. Our results suggest that there are alterations in the cellular localization of C9ORF72 protein as well as in the expression pattern of the isoforms over time. C9ORF72 was detected prenatally, consistent with previous studies looking at the protein in mouse tissue [15]. C9ORF72 was also observed in adult as well as in embryonic and larval stages in zebrafish [2, 15]. A transcription expression study of the mouse ortholog of *C9orf72* found that it was only detectable from P1 in the CNS where it increased gradually until P60 [32]. Koppers [15] suggested these differences could be explained by a failure of the heterozygous LacZ reporter mice used in this study to detect the low levels of gene expression present prenatally.

C9ORF72 isoforms were differentially expressed between the nucleus and cytoplasm. Western blot analysis of nuclear and cytoplasmic protein fractions showed that the 55 kDa band was predominantly nuclear, the 35 kDa band was predominantly cytoplasmic and the 50 kDa band was expressed similarly in both fractions. The expression of mRNA for isoform 1 was higher at P1 than in adult tissue corresponding with higher protein expression of isoform 1 at P1. The higher expression of isoform 1 during these developmental timepoints is consistent with the strong immunohistochemical labeling of C9ORF72 in mouse tissue in postnatal tissue and the localization of C9ORF72 to nuclei at P7. At P56 there was an increase in isoform 2 mRNA compared to P1 and P7. However, this increase in mRNA content was not reflected at the protein level, where isoform 2 protein was significantly higher at early timepoints. These discrepancies may be explained by differences in mechanisms involved in the post-transcriptional regulation, or repression of translation of isoform 2 mRNA in adulthood. A recent study by Xiao and colleagues [37] found differential localization of human C9ORF72 isoforms. The human short isoform (approximately 25 kDa) was localized to the nuclear membrane and the long isoform (approximately 55 kDa) was localized to cytoplasm with diffuse and punctate expression.

The identity of the 110 kDa band labeled by C9ORF72 is unknown. These bands have been observed in previous studies [25] and also in our western blots from primary cell culture (data not shown). As the characteristic labeling of C9ORF72 was reduced following preadsorbtion, we speculate that there is a possibility that it could be a dimer of the 55 kDa band resistant to the reducing agents used in the western blot protocol. Further studies are required to investigate these bands.

Throughout all time points in the current study, C9ORF72 had a punctate pattern of immunolabeling, which is consistent with other studies describing expression of this protein. In mice, synaptogenesis ranges from the first to third weeks of postnatal life [24]. It is therefore plausible that, in the current study, the presence of strongly labeled puncta during this time and reports of diffuse cytoplasmic and punctate labeling from other studies [3, 13, 26, 28, 37] suggest involvement of C9ORF72 at the synapse. Only the 55 kDa form of C9ORF72 was in synaptic-rich fractions in the synaptosome preparations, perhaps indicating a specific role of isoform 1 at synapses. This is also consistent with higher expression of isoform 1 at early postnatal timepoints.

It is unknown why specific populations of cells are vulnerable to degeneration in diseases such as FTLD and ALS. In this study, we showed C9ORF72 expression in neurites and the neuropil. Previous studies have found C9ORF72 within dystrophic neurites within plaques of AD brains and within swollen neurites in the hippocampus of both AD and non-AD brains [26], suggesting that it is present in neurites. Additionally, the protein is observed within swollen axons in the spinal cord ventral gray matter [30]. Motor deficits and abnormal motor neuron axons have been described following knockdown of C9orf72 in zebra-fish [2], although more recent studies in mice have found no effect of complete lack of C9ORF72 on motor function [15]. Our results demonstrate the presence of C9ORF72 as puncta throughout the actin cytoskeleton, and the presence of the protein in synaptic-rich fractions. There are a number of vesicles known to be present in axons including those supplying the synapse, those involved in membrane trafficking and axon outgrowth, and vesicles containing RNA and signaling vesicles [21]. Membrane trafficking is critical for cell survival and defects in transport to the membrane are common hallmarks of neurodegenerative diseases, including FTLD [35]. In a similar line, we recently showed that C9ORF72 is involved in endosomal trafficking via Rab-dependent pathways [7]. When C9ORF72 expression was knocked down, endocytosis and autophagy-related trafficking were inhibited. Human C9ORF72 isoforms have also been shown to interact with nuclear pore complex components, suggesting a possible role in nucleocytoplasmic shuttling [37]. These studies, in combination with our current results related to synaptosome preparations and differential nuclear and cytoplasmic localization, may suggest that C9ORF72 plays a role in trafficking and raises the possibility that failure in such neuronal cellular transport during ageing may be linked to neurodegeneration.

Like many other genetic causes of neurodegenerative disorders, the repeat expansion found in the *C9ORF72* gene is present at birth but does not cause disease until later in life. If haploinsufficiency of the encoded protein, C9ORF72, does contribute to disease then this suggests that it is due to vulnerability caused by altered isoform expression in ageing.

This study has been the first to give a detailed description of the expression of C9ORF72 in mice, including expression over development, and lays a foundation for future studies examining the effects of altering C9ORF72 expression in rodent models, potentially providing insights into how abnormal repeat expansion may be associated with FTLD and ALS. The presence of C9ORF72 within vesicular puncta also warrants further study. Identification of these vesicles could be key to determining the role of this protein within cells.

### Compliance with ethical standards

All applicable international, national, and/or institutional guidelines for the care and use of animals were followed. All procedures performed in studies involving animals were in accordance with the ethical standards of the University of Tasmania.

## Additional files

Additional file 1: Figure S1. Primer efficiency and melting curve analysis. (a). The efficiency of the primer pairs for *C9orf72* isoforms was assessed by plotting the cycle threshold value (Ct) at each concentration against the logarithm of the fold dilution of the sample. The slope of a linear-regression trendline is indicative of primer efficiency. Primer efficiencies were 1.86 for isoform 1 (a i), 1.94 for isoform 2 (a ii) and 1.97 for isoform 3 (a iii). (b) Representative melting curve analysis showing the specific amplification of the *C9orf72* isoform products. Melting peaks (plotted as the negative derivative of fluorescence) revealed peaks at three different temperatures which indicate the identity of amplified *C9orf72* isoforms. (TIFF 24326 kb) Additional file 2: Figure S2. Preadsorbtion with C9ORF72 peptide. (a) P56 tissue from C57/Bl6 mice or (b) 7 DIV cortical neurons cultured from C57/Bl6 mice were labeled with C9ORF72 (sc-138763) antibody or C9ORF72 (sc-138763) antibody preadsorbed with the C9ORF72 peptide (sc-138763 P). Labeling was decreased in both preadsorbed samples. Labeling of puncta (*arrows*) and nuclei (*arrowheads*) with C9ORF72 antibody was present in brain tissue and cultured neurons (panel 1, a, b). In contrast, when labeled with preadsorbed C9ORF72 peptide, there was no nuclei labeling and non-specific puncta present in brain samples (*arrows*, panel 2, a), and in cultured samples there was faint non-specific nuclear labeling and an absence of puncta (*arrowhead*, panel 2, b). Scale bar: a, 12 μm; b, 10 μm. (TIFF 999 kb)
