# Erratum: C9ORF72 expression and cellular localization over mouse development

**DOI:** 10.1186/s40478-015-0253-8

**Published:** 2016-01-04

**Authors:** Rachel A. K. Atkinson, Carmen M. Fernandez-Martos, Julie D. Atkin, James C. Vickers, Anna E. King

**Affiliations:** Wicking Dementia Research and Education Centre, Faculty of Health, University of Tasmania, Hobart, Tasmania Australia; Australian School of Advanced Medicine, Macquarie University, North Ryde, New South Wales Australia

## Erratum

After publication of this article [1], it was noticed there was an error in the Methods section under the subsection: Protein extraction and western blot analysis.

The text including the error is as follows: “Denatured protein samples (15 μg) from each time-point were electrophoresed into 10 % SDS-PAGE gels (BioRad), transferred to PVDF membranes (BioRad) and incubated in primary antibodies overnight (Table 1)”. Instead it should read: “…antibodies, C9ORF72 (1:500, Santa Cruz, sc-138763) and GAPDH (1:7000, Millipore), overnight.”

This error has since been updated in the article [1].
