# Supplementary figures and images for: C9ORF72 expression and cellular localization over mouse development
Source: Acta Neuropathol Commun. 2015 Sep 25;3:59. doi: 10.1186/s40478-015-0238-7 (PMC4582620; doi:10.1186/s40478-015-0238-7)

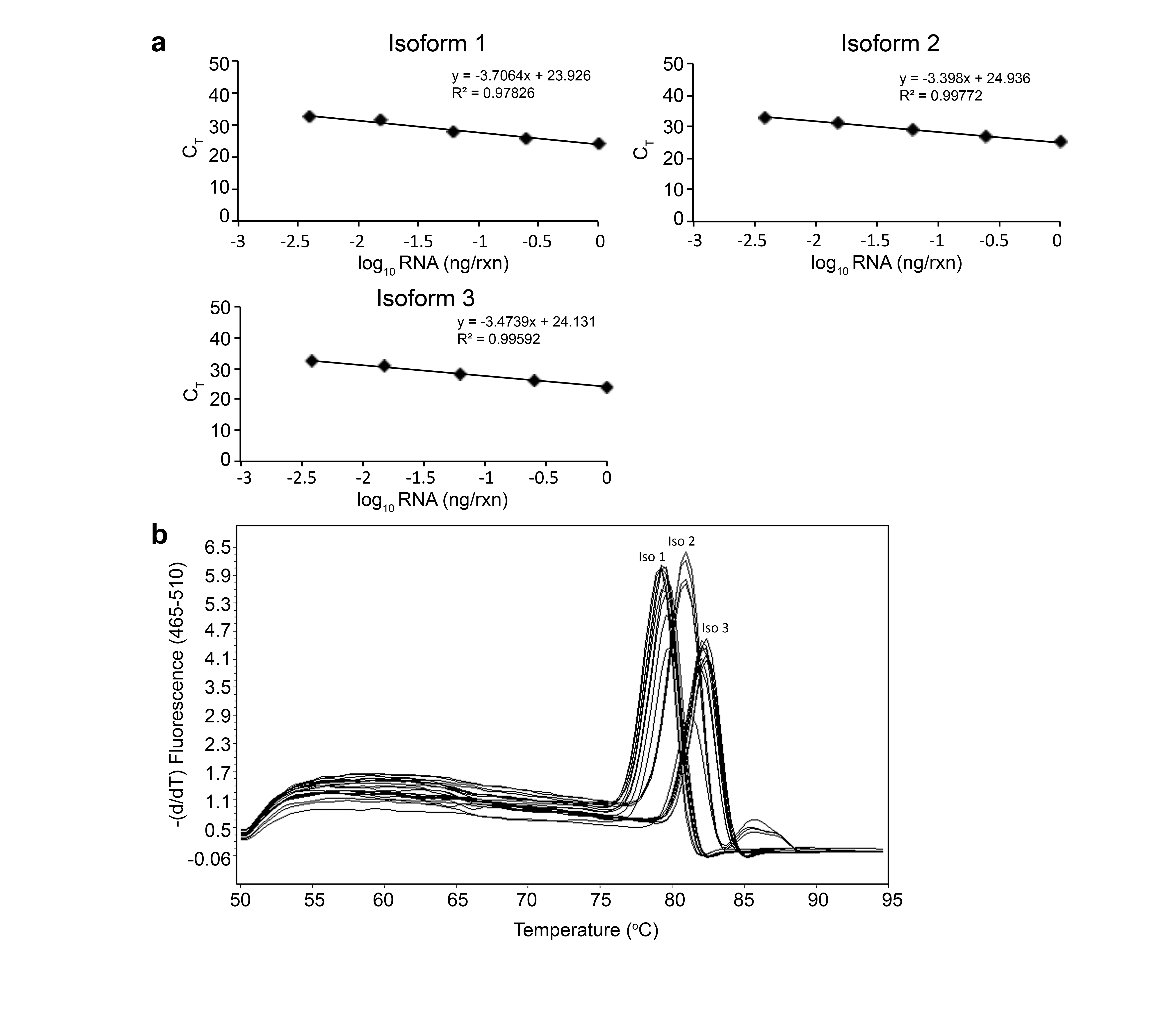

Supplement: Additional file 1: Figure S1. — Primer efficiency and melting curve analysis. (a). The efficiency of the primer pairs for C9orf72 isoforms was assessed by plotting the cycle threshold value (Ct) at each concentration against the logarithm of the fold dilution of the sample. The slope of a linear-regression trendline is indicative of primer efficiency. Primer efficiencies were 1.86 for isoform 1 (a i), 1.94 for isoform 2 (a ii) and 1.97 for isoform 3 (a iii). (b) Representative melting curve analysis showing the specific amplification of the C9orf72 isoform products. Melting peaks (plotted as the negative derivative of fluorescence) revealed peaks at three different temperatures which indicate the identity of amplified C9orf72 isoforms. (TIFF 24326 kb) [file 40478_2015_238_MOESM1_ESM.tif]

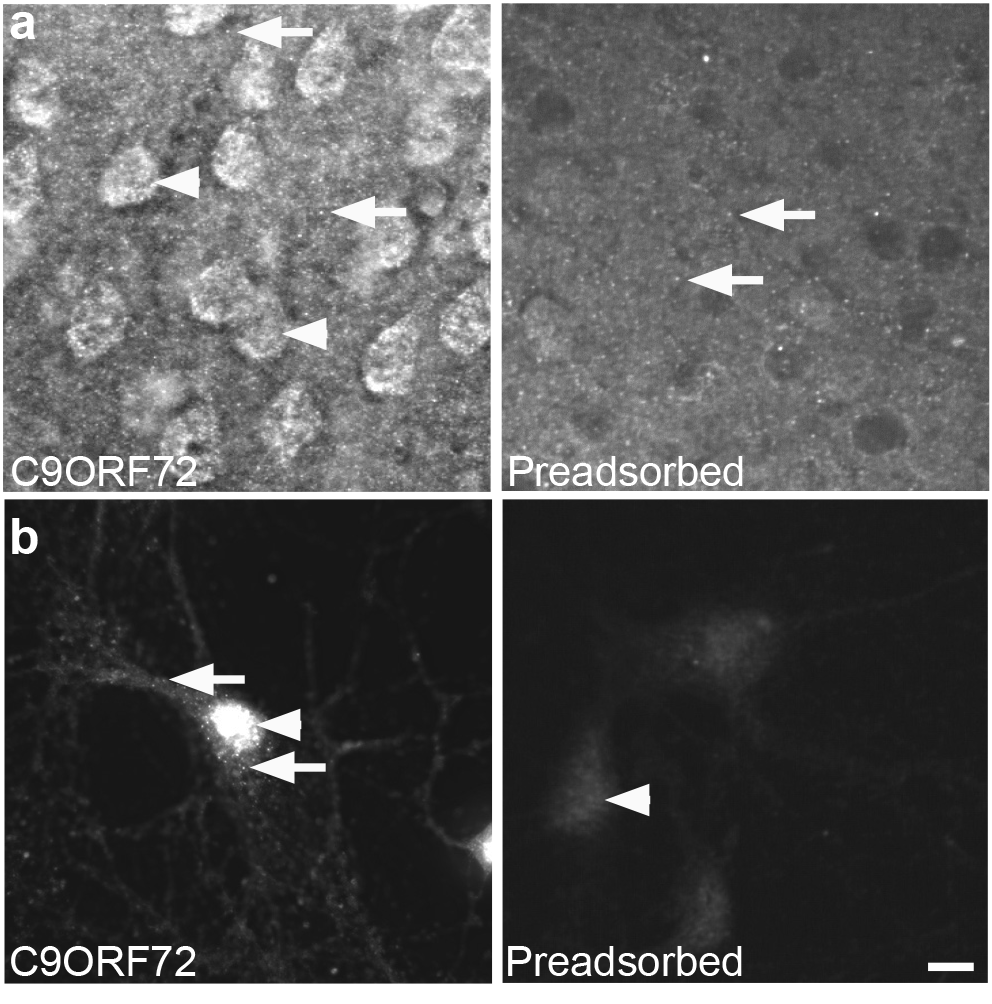

Supplement: Additional file 2: Figure S2. — Preadsorbtion with C9ORF72 peptide. (a) P56 tissue from C57/Bl6 mice or (b) 7 DIV cortical neurons cultured from C57/Bl6 mice were labeled with C9ORF72 (sc-138763) antibody or C9ORF72 (sc-138763) antibody preadsorbed with the C9ORF72 peptide (sc-138763 P). Labeling was decreased in both preadsorbed samples. Labeling of puncta (arrows) and nuclei (arrowheads) with C9ORF72 antibody was present in brain tissue and cultured neurons (panel 1, a, b). In contrast, when labeled with preadsorbed C9ORF72 peptide, there was no nuclei labeling and non-specific puncta present in brain samples (arrows, panel 2, a), and in cultured samples there was faint non-specific nuclear labeling and an absence of puncta (arrowhead, panel 2, b). Scale bar: a, 12 μm; b, 10 μm. (TIFF 999 kb) [file 40478_2015_238_MOESM2_ESM.tif]
